# Association of Angiopoietin-2 and Dimethylarginines with Complicated Course in Patients with Leptospirosis

**DOI:** 10.1371/journal.pone.0087490

**Published:** 2014-01-30

**Authors:** Alexander Lukasz, Bodo Hoffmeister, Barbara Graf, Benno Wölk, Karsten Noeckler, Stefanie M. Bode-Böger, Johannes Hadem, Sven Pischke, Jan T. Kielstein

**Affiliations:** 1 Department of Nephrology & Hypertension, Hannover Medical School, Hannover, Germany; 2 Department of Medicine D, Division of General Internal Medicine, Nephrology, and Rheumatology, University Hospital Muenster, Muenster, Germany; 3 Department of Infectious Diseases and Pulmonology, Charité University Medicine, Berlin, Germany; 4 Department of Microbiology and Hygiene, Charité University Medicine, Berlin, Germany; 5 Institute for Virology, Hannover Medical School, Hannover, Germany; 6 Bundesinstitut für Risikobewertung, Abteilung für Biologische Sicherheit, Berlin, Germany; 7 Institute for Clinical Pharmacology, Otto-von-Guericke University, Magdeburg, Germany; 8 Department of Gastroenterology and Hepatology, Hannover Medical School, Hannover, Germany; University of São Paulo School of Medicine, Brazil

## Abstract

Leptospirosis is one of the most relevant zoonosis worldwide and a potentially life-threatening infectious disease. While it is frequent in tropic regions, it is uncommon in European industrialized countries. Angiopoietin-2 (Angpt-2) and asymmetric and symmetric dimethylarginine (ADMA and SDMA) are markers of endothelial activation and systemic inflammation. These parameters have been studied recently in the context of sepsis and MODS showing potential to determine disease severity and outcome specific parameters like acute kidney injury (AKI) and survival. These biomarkers were measured in 13 patients with leptospirosis. High levels of Angpt-2 were statistically significant associated with a complicated clinical course with occurrence of AKI, Sepsis and intensive care unit treatment. ADMA was significantly associated with occurrence of AKI and ICU treatment whereas SDMA was associated with AKI. Therefore these endothelial markers may serve as additional tools for risk stratification in these patients.

## Introduction

Leptospirosis is one of the most relevant zoonosis worldwide. It is frequent in tropic regions but uncommon in European industrialized countries [1]. While most of the infected exhibit a subclinical or very mild course, approximately 5–10% of patients suffer from potentially fatal disease due a multi-organ dysfunction syndrome (MODS) also known as Weil’s disease. It comprises of the triad jaundice, acute kidney injury (AKI) and hemorrhages and is associated with mortality rates of 5% to 15% [2].

Different scores have been evaluated to predict the clinical severity of leptospirosis [3]. However, there is no simple clinical or laboratorial parameter available that allows assessing, let alone predicting the clinical course at the time of diagnosis. Hence, there is an ongoing quest for markers able to differentiate between severe courses requiring treatment in the intensive care unit and moderate courses requiring a lower level of care.

Angiopoietin-2 (Angpt-2) and asymmetric and symmetric dimethylarginine (ADMA and SDMA) are novel markers of endothelial activation and systemic inflammation. These parameters have been studied recently in the context of sepsis and MODS showing potential to determine disease severity and outcome specific parameters like length of stay (LOS), acute kidney injury (AKI) and survival [4], [5]. Despite their increasing availability and standardization these markers are not yet used in clinical routine. We aimed to evaluate these parameters as potential predictors of the clinical course in a retrospective cohort of patients with leptospirosis.

## Methods

### Patients and Study Design

A total of 13 patients with confirmed leptospirosis treated at German tertiary-care university hospitals (Berlin, n = 8; Hannover n = 5) from May 2001 to July 2010 were included. The Ethics Committee of the Hannover Medical School and Charité university hospital waived the need for written informed consent from the patients. According to the principles of these two Committees neither ethical approval nor informed consent is necessary, if I) data acquisition was retrospective observational, II) data were anonymized, and III) the investigation relied on measurements and data acquisition applied as part of routine care.

### Definitions

According to the German top level health authority, the Robert Koch Institute (RKI), and international guidelines the diagnosis was based on: at least one clinical symptom (fever, renal dysfunction, jaundice, cough or dyspnea, meningitis, hemorrhages, or myocarditis) in conjunction with laboratory confirmation by seroconversion, positive culture, positive polymerase chain reaction (PCR), complement fixation testing (CFT), or microscopic agglutination testing (MAT) according to laboratory standards of the participating institutions as described previously [6].

### Sampling and Quantification of Angiopoietin-2, ADMA and SDMA

Blood samples for measurement of serum Angpt-2, ADMA, SDMA and routine chemistry were drawn and immediately cooled on ice. Supernatants were stored at –80°C until further use.

Plasma concentrations of Angpt-2 were measured by an in-house enzyme-linked immunosorbent assay (ELISA) as previously described in detail [7]. Serum ADMA and SDMA were assessed using high-performance liquid-chromatography-tandem mass-spectrometry [8]. In brief interassay imprecision was 4.6% for Angpt-2, 3.8% for ADMA and 3.9% for SDMA, respectively. All other measurements were done with routine laboratory tests using certified assay methods. Twenty-seven apparently healthy volunteers served as controls (age between 25 and 73 years).

### Statistical Analysis

One-way ANOVA and Bonferroni’s multiple comparison test was applied for comparison of quantitative variable. Two-tailed *P* values <0.05 were considered statistically significant. Correlations between variables were assessed by linear regression. Data analysis was performed using SPSS (SPSS Inc, Chicago, Illinois, USA). Figures were prepared using the GraphPad Prism (GraphPad Prism Software Inc, San Diego, California, USA).

## Results

### Patient Characteristics

In total, 13 adult patients, 3 women (23%) and 10 men (77%) between 27 and 59 years of age (median age 39 years) were included in this study.

Time from onset of symptoms to presentation was 3 days in median, range 1–18 days, and the time required for establishment of the diagnosis was 4 days in median, range 2–14 days. Most patients with leptospirosis acquired the disease at the tropics (n = 10 (77%)). Blood for the quantification of Angpt-2, ADMA and SDMA was collected a median of 2 days (range 1–3 days) after admission. The median duration of hospitalization was 10 days (range 9–12). Patient characteristics and outcomes are shown in Table 1.

**Table 1:** Demographic, clinical and laboratory characteristics and outcomes of patients.

Variable	Total
**Demographics**	
Number of patients (n)	13
Age (years, median (IQR))	39 (36–46)
Female sex (n, %)	3 (23)
Time from onset of symptoms to presentation (days, median, (range))	3 (1–18)
Time for confirmation of diagnosis (days, median, (range))	4 (2–14)
Collection of samples (days, median (range))¥	2 (1–3)
**Risk Factors for infection** (n, %)	
Travelling to the tropics*	10 (77)
Water contact*	11 (85)
Direct contact to animals*	3 (23)
**Confirmation of diagnosis**	
Seroconversion (n, %)	13 (100)
Microscopic agglutination testing (n, %)	1 (8)
Polymerase chain reaction (n, %)	1 (8)
**Laboratory data on admission (median (IQR))**	
Hemoglobin (g/dl)	14.5 (12.1–15)
Platelets (10^6^/µl)	131 (45–156)
Leukocytes (10^3^/µl)	7.6 (5.58–8.53)
Creatinine (µmol/l)	102 (86–187)
CRP (mg/l)	124 (54.6–225.3)
Bilirubine (µmol/l)	39.4 (17.4–54.5)
ALT (U/l)	53 (28–87)
CK (U/l)	147.5 (120–616.5)
**Worst laboratory data (median (IQR))**	
Hemoglobin (g/dl)	11.3 (10–12.5)
Platelets (10^6^/µl)	83 (22–131)
Leukocytes (10^3^/µl)	9.6 (6.88–12.05)
Creatinine (µmol/l)	166 (86–232)
CRP (mg/l)	162 (82–232.6)
Bilirubine (µmol/l)	43.8 (29.1–55.3)
ALT (U/l)	91 (57–162)
CK (U/l)	156 (87–716)
**Outcome**	
Length of hospital stay (days, median, (IQR))	10 (9–12)
Need of intensive care treatment (n, %)	6 (46)
Length of ICU stay (days, median, (IQR))	2.5 (1.3–5.3)
Acute Kidney Injury (n, %)	7 (54)
Sepsis, total (n, %)	8 (62)
Sepsis, present on admission (n, %)	7 (54)
Need of mechanical ventilation (n, %)	1 (8)
death (n, %)	0 (0)

¥ in days after admission.

* in a direct context with the onset of the infection (<3 weeks).

Abbrevations: CRP = C-reactive protein; ALT = alanine transaminase; CK = creatine kinase; ICU = intensive care unit.

### Angiopoietin-2, ADMA and SDMA Levels in Relation to Clinical Complications of Leptospirosis

There was a significant association between C-reactive protein and Angpt-2 (r^2^ = 0.56, p = 0.004), but not between ADMA, SDMA and C-reactive protein, respectively (data not shown).

### Occurrence of Sepsis

**Figure 1:**
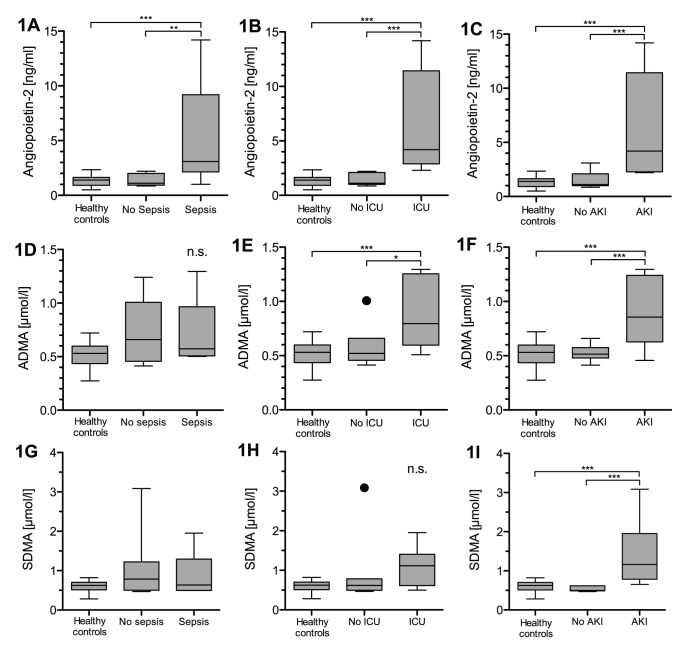
Box plots of results in healthy controls and study patients. Circulating Angpt-2 in septic patients (n = 8) (A), patients with need of intensive care treatment (n = 6) (B) and patients developing AKI (n = 7) (C). Circulating ADMA in septic patients (D), patients with need of intensive care treatment (E) and patients developing AKI (F). Circulating SDMA in septic patients (G), patients with need of intensive care treatment (H) and patients developing AKI (I). (n.s. = not significant, * = p<0.05, ** = p<0.001, *** = p<0.0001).

Patients with leptospirosis and sepsis (according ACCP/SCCM definition) had significant higher levels of circulating Angpt-2 levels compared to patients without sepsis and healthy controls (5.2 vs. 1.4 vs. 1.3 ng/ml, p<0.001) (Fig. 1(a)). ADMA and SDMA levels were not different between patients with and without sepsis and healthy controls (Fig. 1(d) and (g)).

### Need of Intensive Care Treatment

Patients with leptospirosis treated in the intensive care unit had significantly higher levels of circulating Angpt-2 compared to patients without need of intensive care treatment as well as healthy controls (6.4 vs. 1.4 vs. 1.3 ng/ml, p<0.0001) (Fig. 1(b)). While ADMA was different in patients with need of intensive care treatment compared to those without and healthy controls (0.88 vs. 0.59 vs. 0.53 µmol/l, p<0.05, p<0.0001, respectively) (Fig. 1(e)), SDMA was not significantly different (Fig. 1(h)).

### Occurrence of Acute Kidney Injury

Circulating Angpt-2, ADMA and SDMA were also different in patients with AKI and those without as well as healthy controls (Angpt-2: 6.3 vs. 1.6 vs. 1.3 ng/ml; ADMA: 0.89 vs. 0.52 vs. 0.53 µmol/l; SDMA: 1.4 vs. 0.53 vs. 0.60 µmol/l; p<0.0001, respectively) (Fig. 1(c), 1(f), 1(i)).

## Discussion

This is the first study investigating circulating Angpt-2, ADMA and SDMA levels in patients with confirmed leptospirosis. Our data indicate that these markers can be used to identify patients at risk with a complicated course of the disease.

Evidence is increasing that endothelial activation in the sense of a disruption of the endothelial cell layer may be an important pathophysiologic mechanism in leptospirosis [9]. Recent studies underlined the potential of Angpt-2 as a marker and mediator of endothelial activation. In vitro studies indicated that high Angpt-2 levels can cause endothelial activation with a consecutive disruption of the endothelial cell layer, which supports the role of Angpt-2 as a mediator of endothelial activation [5]. Interestingly, plasma exchange, which had been used for severe cases of leptospirosis [10] has been shown to reduce Angpt-2 in the setting of thrombotic microangiopathy [11]. Bacterial toxins such as lipopolysaccharides (LPS) are potent trigger for the release of Angpt-2. The significant correlation between the established marker CRP and Angpt-2 in this cohort underlines the role of Angpt-2 as an inflammation marker.

ADMA, a powerful marker for long-term cardiovascular risk [12] decreases in the state of acute inflammation [13]. Indeed the abrupt increase of inflammatory mediators is accompanied by a simultaneous decrease in plasma ADMA [14], hence we did not expect an increase in ADMA levels. ADMA was however elevated in critically ill patients, reflecting the dysfunction of two key organs excreting and/or metabolizing ADMA, i.e. the kidney and the liver. SDMA, the structural isomer of ADMA with no direct effect on NOS activity that is almost exclusively removed via renal excretion was able to identify patients with acute kidney injury, confirming previous studies showing that it increase already within 6 hours after a marked change in glomerular filtration rate [15].

The very nature of the study does not allow firm conclusions. However, at least from a biomarker point of view Angpt-2, ADMA and SDMA may be helpful tools to diagnose endothelial activation and detecting patients in which leptospirosis is accompanied by severe hepatic and renal involvement. Our novel results, obtained in a rather small number of patients with a rare disease should be prospectively tested in larger cohorts. This study is not able to proof the value of Angpt-2, ADMA and SDMA for the prediction of the clinical course of leptospirosis in general. However based on our data one can hypothesize that these markers could be used for the risk management of leptospirosis in the future. Moreover, our results help to understand the beneficial effects of interventions like plasma exchange, which removes Angpt-2. Moreover as pharmacological modifiers of Angpt-2 and ADMA are tested in preclinical studies our observation might be important to develop new therapeutic approaches for critically ill patients with leptospirosis.
